# Eco-Environmental Aspects of COVID-19 Pandemic and Potential Control Strategies

**DOI:** 10.3390/ijerph18073488

**Published:** 2021-03-27

**Authors:** Rashid Nazir, Jawad Ali, Ijaz Rasul, Emilie Widemann, Sarfraz Shafiq

**Affiliations:** 1 Department of Environmental Sciences, Abbottabad Campus, COMSATS University Islamabad, University Road, Abbottabad 22060, Pakistan; rashidnazir@cuiatd.edu.pk; 2 Department of Biotechnology, Abbottabad Campus, Tobe Camp, COMSATS University Islamabad, University Road, Abbottabad 22060, Pakistan; jawadbiotech@gmail.com; 3 Plant Virology Section, Department of Bioinformatics and Biotechnology, Government College University Faisalabad, Faisalabad 38000, Pakistan; i.rasulrug@gmail.com; 4 Department of Biology, University of Western Ontario, London, ON N6A5B7, Canada; ewidema4@uwo.ca; 5 Department of Anatomy and Cell Biology, University of Western Ontario, London, ON N6A3K7, Canada

**Keywords:** COVID-19, virus, environment, ecology, viral spread, disease outbreak, SARS-CoV-2

## Abstract

A new coronavirus-strain from a zoonotic reservoir (probably bat)—termed as severe acute respiratory syndrome coronavirus 2 (SARS-CoV-2)—has recently claimed more than two million deaths worldwide. Consequently, a burst of scientific reports on epidemiology, symptoms, and diagnosis came out. However, a comprehensive understanding of eco-environmental aspects that may contribute to coronavirus disease 2019 (COVID-19) spread is still missing, and we therefore aim to focus here on these aspects. In addition to human–human direct SARS-CoV-2 transmission, eco-environmental sources, such as air aerosols, different public use objects, hospital wastes, livestock/pet animals, municipal wastes, ventilation facilities, soil and groundwater potentially contribute to SARS-CoV-2 transmission. Further, high temperature and humidity were found to limit the spread of COVID-19. Although the COVID-19 pandemic led to decrease air and noise pollution during the period of lockdown, increased use of masks and gloves is threatening the environment by water and soil pollutions. COVID-19 badly impacted all the socio-economic groups in different capacities, where women, slum dwellers, and the people lacking social protections are the most vulnerable. Finally, sustainable strategies, waste management, biodiversity reclaim, eco-friendly lifestyle, improved health infrastructure and public awareness, were proposed to minimize the COVID-19 impact on our society and environment. These strategies will seemingly be equally effective against any future outbreak.

## 1. Introduction

Although they are non-living particles, viruses are the parts and particles of all life on earth i.e., ranging from bacteriophages in microbes to coronaviruses in mammals; ultimately contributing significant impacts on various aspects of human as well as on other types of life [1]. The environment where living and non-living things interact with each other for the overall objectivity, is termed an ecosystem [1] and its rich biodiversity is the main source of overall functionality. An ecosystem thus requires a range of different activities for its optimum performance, and these varied activities need different living entities for their execution. Noticeably, the loss of any living entity means the loss of that particular activity (being done by the life) and ultimately disturbing the optimum functioning of the ecosystem [2]. On the other side, humans have exploited natural resources in a way that has deteriorated nature via urbanization and other anthropogenic activities for thousands of years. As a result of these human-induced changes, biodiversity has been seriously compromised, and the natural imbalance of an ecosystem in return adversely affects human living as well. In order to adapt to this consistently changing environment and as a survival of the fittest strategy, genetic material of organisms may modify itself (most likely by mutations) and consequently, the mutated genotype (and associated consequent morphotype) makes it more suitable in a newly set challenged environment (habitat) [3]. Along these similar lines of genetic evolution, most likely, the driving forces induced by either habitat, host, or environment may cause mutations (over time) to viruses. Severe acute respiratory syndrome coronavirus (SARS-CoV; 2002–2003 outbreak) and Middle East respiratory syndrome (MERS; 2012 outbreak) are the examples of such mutations and the resultant viruses have been deadly pathogens to the human health.

Recently, the novel COVID-19 causing SARS-CoV-2 virus has probably evolved from coronavirus SARS, being better to infect (and survive in) humans [4]. Consequently, the COVID-19 infection turned into a pandemic, and the whole world was shut down, suspended its major activities and restricted human life to necessities only. In this context, the host-to-human and human-to-human direct transmission through respiratory droplets are repeatedly reported as the direct sources of COVID-19 spread [5]. Social distancing and lockdown, in most parts of the world, somehow limit the daily new cases. However, lockdown cannot be a sustainable solution and human life has to continue in a new normal. This, therefore, necessitates considering the other transmission possibilities as well, particularly the human lifestyle and associated environmental contributions. Some preliminary evidence shows that environmentally facilitated corona viral transmission may be possible, specifically, that COVID-19 patients could get the virus through connection with abiotic built-environmental surfaces [6]. Therefore, the purpose of this synthesis is to evaluate different human-associated environmental aspects and factors which may facilitate or restrict the COVID-19 spread, and to summarize the impacts of COVID-19 on our socio-economic infrastructure and environment.

## 2. Origin and History of COVID-19

The novel coronavirus was isolated in Wuhan, China in December 2019, whereas another study reported this virus to be present in Barcelona wastewater during March 2019 [7], suggesting that the novel coronavirus was already in the environment before the Wuhan outbreak. The histories and information gathered from the early patients traced this virus back (and even linked in some ways) to the Wuhan local animal (seafood) market, suggesting a direct food-based spread to human [8]. These observations suggest that animals are the likely origin of this novel coronavirus. The World Health Organization (WHO) named the disease coronavirus disease 2019 (COVID-19), while the International Committee on Taxonomy of Viruses (ICTV) named the causal agent (in February 2020) severe acute respiratory syndrome coronavirus 2 (SARS-CoV-2) [9]. The genetic analysis of the novel SARS-CoV-2 has shown 88% homology with bat-derived SARS-CoV, and about 50% of the genome is matched with MERS-CoV [6]. A systematic comparison between SARS-CoV, MERS-CoV, and SARS-CoV-2 is summarized in Table 1.

## 3. Eco-Environmental Sources of COVID-19 Transmission

The most direct transmission of coronavirus SARS-CoV-2 between humans can occur through coughing and sneezing by an infected person [8]. The novel coronavirus (SARS-CoV-2) multiplies in the airways and lung cells of an infected person. Additionally, when an infected cell dies, the viruses are released into the thin layer of fluid lining the airway and lung, and consequently the virus gets into the nasal cavity and also into the saliva and spit of the oral cavity [10]. In this context, COVID-19 infected people may shed more virus particles into the environment.

**Figure 1:**
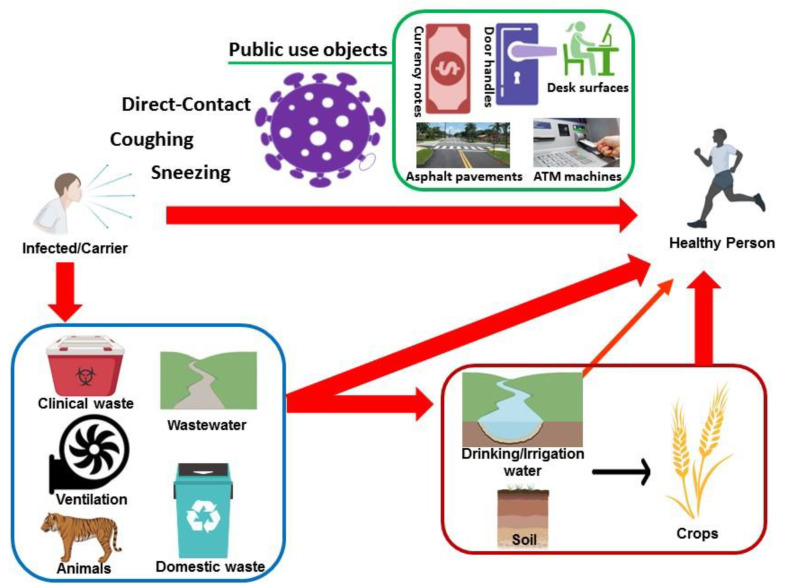
Schematic depiction of the eco-environmental aspects for potential COVID-19 (direct and indirect) human-to-human spread through (in) the human whereabouts.

Consequently, SARS-CoV-2 can also spread indirectly from one individual to another through the environment. Such indirect transmission might be possible through oral mucosa [11]. The novel coronavirus (SARS-CoV-2) has similar biological properties compared to the previously discovered SARS-CoV which had been identified in the excretions from the sputum, feces, urine, and blood of the infected patients [12]. SARS-CoV can survive for about 15 days in the blood, 10 days in the urine, and about 5 days in the feces and sputum at 24 °C. Whereas on the surface of plastics, gauze, glass, and filter paper, the virus can survive for about 3 days at room temperature [13]. All this evidence suggests a possible connection between eco-environmental factors and COVID-19 spread. Some of these eco-environmental (indirect) sources of COVID-19 spread are summarized in Figure 1.

### 3.1. Air Aerosols

Viruses are very small in size, approximately 20–300 nm, and they are generally transported in the air from one place to another through respiratory droplets [14]. Previous epidemiological studies suggested that the viral transmission inside and between adjacent houses [15] may occur through the contaminated aerosols generated and present in the air. In addition to the transmission of SARS-CoV-2 through sneezing/coughing, The National Academics Standing Committee on Emerging Infectious Diseases and 21st Century Health Threats has considered the transmission of SARS-CoV-2 even through the conversation (National Academics of Sciences, 2020). So far it has not been confirmed that the coronavirus identified from air sampling is viable and can infect others [16]. It much likely depends on the time of exposure and number of the virus particles. Biological risk of COVID-19 inhalation transmission is very high while carrying dental procedures, owing to the use of handpieces under irrigation, which supports the diffusion of aerosol particles of saliva, blood, and secretions [17].

There are many other factors responsible for the transport of viral particles from one place to another in the form of air aerosols. The distance traveled by the infection droplets when a person coughs/sneezes is directly dependent on the wind speed in an open environment. The droplets from an infected person will fall on the ground after a very short distance in the absence of surrounding wind speed. At a wind speed of 4–15 km/h, the saliva droplets can travel up to 6 m, which means that the social distancing of 2 m (the largely recommended remedy) may not be able to prevent the individuals from becoming infected [18]. It is, thus, necessary to consider all the scenarios that may allow the transmission to longer distances. The current experimental air sampling technologies used for the detection and determination of the virus in aerosol particles are also not accurate enough and have many drawbacks [19]. Therefore, the more advanced technologies and research should be carried out to accurately assess the presence of viable virus particles in air samples.

### 3.2. Public Use Objects

The movement of people from one place to another inside the built environment leads to direct or indirect contact with the surfaces around them. The infected/diseased person may start spreading the virus before, during, as well as after the development of symptoms, contaminating the surfaces everywhere [20]. That is why these viral particles can be found on the door handles, desk surfaces, currency notes, ATM-machines, and other public use objects. It has been reported that the persons infected with COVID-19 shed viral particles from their bodily secretions, such as saliva or nasal fluid, when they are talking, coughing, sneezing, and/or vomiting. These viral particles can infect the non-living objects in their surroundings [21,22]. SARS-CoV-2 viral particles have also been detected from various objects inside a COVID-19 patient’s room and also from the toilet area but this study was performed on small sample size and region with lesser cases of COVID-19 [21] and thus needs further verifications. During the outbreak of MERS-CoV, almost every touchable surface inside a hospital dealing with MERS patients was contaminated with the viral particles [23]. Similarly, an investigation of a hospital room with an isolated COVID-19 patient, verified widespread environmental contamination [24]. Though data on the transmission dynamics of COVID-19 is currently emerging, it appears possible that SARS-CoV-2 can hypothetically persist on fomites extending from a couple of hours to 5 days depending on the material under subject [25].

Moreover, the data described on the endemic human coronavirus strain (HCoV) 229E indicate that the virus can remain infectious from 2 h to 9 days on different surfaces [25]. The frequently touched surfaces and contamination in healthcare settings—and/or elsewhere—may serve as hotspot for the transmission of viral particles from one individual to another [25]. There are no data available on the transmission of coronaviruses from polluted materials to hands. Contrary to that, in the case of the influenza A virus, it has earlier been reported that only 5 s of contact can transfer 31.6% of the viral load from the contaminated surface to the human hands [26]. This transfer efficacy was lesser (1.5%) with parainfluenza virus 3 during the same 5 s interaction [27]. Therefore, the potential of human hands’ infection from viral contaminated surfaces is very important for the disease spread and for its control. In this specific situation, an observational study has defined that humans (students in their case) touch their face with their own hands 23 times per hour on average. Of all these face touches, 44% involve contact with the mucous membrane, while the remaining 56% of contacts are with the non-mucosal areas. The mucous membrane touches involve contact mostly to the mouth (36%), nose (31%), eyes (27%), and the remaining 6% are a combination of all these [28]. So far, the viral load of SARS-CoV-2 on non-living objects is not very well explored for COVID-19, but active disinfection of surfaces in the surroundings of confirmed COVID-19 patients would help to control the spread.

### 3.3. Livestock and Pet Animals

Livestock and pet animals are the reservoirs for the transmission of diverse pathogenic viruses to humans. The available sequencing data shows that all the human coronaviruses have a zoonotic origin. SARS-CoV, SARS-CoV-2, MERS-CoV, HCoV-NL63, and HCoV-229E are reported to be initiated in bats, while HCoV-OC43 and HKU1 are supposed to be originated from rodents [29,30]. In this scenario, domestic animals are of greater importance because they act as an intermediate in the transmission of viruses from their natural hosts into humans. It is found that SARS-CoV-2 replicates poorly in dogs, pigs, chickens, and ducks, but ferrets and cats are indulgent to infection. Moreover, cats are susceptible to airborne transmission as well [31]. It is also shown that SARS-CoV-2 is transmitted efficiently via direct as well as via the air between ferrets, 1–3 days and 3–7 days after getting exposed, respectively. The study thus showed experimental evidence of robust transmission of SARS-CoV-2 via aerosols [32].

A small number of pet cats and dogs, who were in close contact with COVID-19 patients, have been reported to be infected with SARS-CoV-2. Similarly, several lions and tigers at a New York zoo and mink (which are closely related to ferrets) on multiple farms in Netherland, Denmark, Spain, and the United States also tested positive for SARS-CoV-2. Public health officials believe that the lions, tigers, and mink became sick after being exposed to employees who were infected with SARS-CoV-2 [33]. These observations from public health officials must be scientifically evaluated, so that human–animals transfer of SARS-CoV-2 can also be controlled to protect the wildlife as well.

### 3.4. Municipal Wastewater

Domestic as well as animal husbandry waste, with possible viral contaminations from household and farm waste, normally end up in the wastewater. The virus-particles have also been found in the feces of COVID-19 patients [34], making it a hopeful contender for the wastewater-based epidemiology (WBE). During the 2002–2003 SARS-CoV epidemic, virus particles were detected in Chinese hospital wastewater [35].

In continuation, many current studies have reported the presence of SARS-CoV-2 in wastewater. Even before the onset of COVID-19 disease, Spanish researchers found this virus in their samples of Barcelona wastewater during March 2019 [7]. During the SARS-CoV-2 outbreak in China, viral RNA was found in the feces of the patients [21], and another study reported the presence of SARS-CoV-2 RNA in the wastewater discharge from a (COVID-19 designated) hospital in China, which indicates the potential of virus to contaminate the drainage system [36]. It is further reported that the wastewater system can enable the airborne transmission of SARS-CoV-2 under specific conditions [37]. Another related study, performed in Massachusetts, tested the wastewater from major urban treatment facilities and reported the presence of SARS-CoV-2 in high titers by using RT-qPCR technology. The identity of the PCR product was also confirmed by direct DNA sequencing. Viral titers were observed significantly higher than expected, based on clinically confirmed cases in Massachusetts, as of 25 March 2020 [8]. The scientific reason for this discrepancy is not yet clear, but the presence of human epithelial cells (excreted through feces, urine, and/or oral spits) in wastewater may facilitate the viral replication and consequently the increased abundance in this particular habitat. In support of this viewpoint, virus particles have sometimes been reported to be present in the feces of infected personnel, though rarely present in blood [38]. Further investigations in this environmental context are still needed.

### 3.5. Hospital/Clinical Waste

The waste generated during the COVID-19 pandemic by hospitals is approximately 6 times more than the normal situations. Moreover, inappropriate treatment of the medical and non-medical material used by the patients and mucosa exposure can also increase the risks of COVID-19 transmission to other healthy people. When patients and healthcare providers use personal protective equipment (PPEs) like masks, gloves, suits, and other items like needles, sharps, gauze, and different types of other pathological wastes; all these used PPEs and other items may get contaminated by the virus. During the COVID-19 pandemic, the overall production of clinical waste increased [39], which can speed up the disease spread and consequently may cause a greater number of infections in the health care staff and other patients [40]. Further, when disposed of into the open environment (without appropriate treatment), this clinical waste may serve as hotspot for the transmission of the virus into the surrounding environment and ultimately to the nearby human community as well. In Wuhan China, officials had to construct a new medical waste plant and to deploy 46 mobile waste treatment facilities due to the higher amount of waste produced by the hospitals during the outbreak. Hospitals are also the major sources of contagious secretions resulting from diagnostic, laboratory, and research activities. Clinical waste includes various active components of drugs and metabolites, chemicals, pharmaceutical remains, radioactive markers, iodinated contrast media, etc. [41]. Therefore, the release of toxic hospital wastes into the environment without prior treatment could cause severe harmful effects on the environment and the general health of the people [42]. Therefore, workers in municipal solid waste and wastewater management are at high risk of getting SARS-CoV-2. All these observations suggest that clinical waste should be properly disposed in the presence of trained workers.

### 3.6. Airflow and Ventilation

Ventilation and associated airflows have been reported to have a specific role in COVID-19 spread, particularly in-built environments. Therefore, the narrow and/or closed places with poor airflow and ventilation, are at higher risk for the accumulation of COVID-19 particles and subsequent transmission to the healthy people therein. The aerosol transmission is also possible through burst ventilation, particularly when a healthy individual is exposed to elevated aerosol concentrations for a larger period in closed spaces [43]. The recent data obtained from the SARS-CoV-2 spread in China reveals that the virus-spread is initially limited to the family members, health care providers, and other close contacts [44], particularly the people living in a closed environment and tentatively sharing the same ventilation facility.

In Guangzhou China, 10 persons from 3 families (A–C) who had eaten at the same air-conditioned restaurant were infected with COVID-19 from 26 January to 10 February 2020 [45]. Out of these families, family A traveled to Wuhan (center of SARS-CoV-2 outbreak) and arrived back to Guangzhou. The suspected case in family A (A1) had lunch with other 3 family members at the restaurant in which family B and C were sitting on the tables near them. After that day, patient A1 experienced fever and cough (symptoms of COVID-19) and was hospitalized. Since then, a total of 9 other persons who were present in the same restaurant and were close to the patient A1 became infected with COVID-19. Out of these 9, 4 were family A members, 3 from family B, and 2 were from family C. The only predicted source of persons in family B and C contracting the virus was the patient A1 at the restaurant [45]. The outlet and inlet for the air conditioner were located above the tables where the families were seated, which indicates that strong airflow have propagated the viral droplets and therefore the people sitting nearby became infected with SARS-CoV-2.

Another research conducted in Hong Kong indicated that human-to-human transmission of SARS-CoV-2 is likely to be influenced by airflow as well as ventilation [46]. They performed a pilot experiment for the examination of exhaled air from a confirmed COVID-19 patient with a medium level of viral load in respiratory specimens. The results demonstrated that viral RNA was absent in the air samples but present in the environmental samples. However, the conclusion cannot be drawn on a single patient and further research is required to explore the airborne transmission of SARS-CoV-2.

### 3.7. Soil and Groundwater

Being the ultimate sink of various terrestrial pollutants, soil may receive the viral contamination via different routes from various source points. Through soil, viruses may even transport to surface as well as underground water, causing major concerns to nearby human community. As per records, human life has suffered from many epidemics of infectious diseases due to the consumption of contaminated (ground) water, and soil has acted as a vector and even source of human pathogenic agents [47]. Due to the land disposal practices of sewage and sludge, there is a high possibility that soil-borne diseases in the human population may increase in the future. For instance, in some countries, untreated domestic wastewater is used for irrigation to crops, which poses a high risk to the working farmers as well as to those consumers who consume the raw food irrigated with such potentially contaminated wastewater [48]. Occurrence and survival of viruses in the soil may thus possess varied public health implications, but there is very little knowledge available on the fate of viruses, particularly COVID-19, in soil due to the complex methodologies for the extraction of viable virus particles from soil samples.

Groundwater contains fewer microbial populations than surface water. However, the role of groundwater cannot be ignored in causing different diseases because the underground water causes approximately half of the waterborne disease-outbreaks [49]. Most of these outbreaks were associated with a small water system having a minimal/limited monitoring system for the potential problematic microbes [49]. Via passing through soil, the infiltrating pathogens can enter underground water from septic tanks, leaking sewers, and the leakage of livestock manure tanks. Human enteric viruses are one of the microbial contaminants, which are of greater concern in a well-water system. These viruses are shed in a larger number in human stools and are thus associated with disease outbreaks [50]. The previously studied coronaviruses are reported to rapidly die in wastewater with almost 99% reduction in two to three days, but they may survive for weeks in groundwater and, thus, present the potential problem for human health [51]. Further scientific investigations are still needed to evaluate the fate and dynamics of the SARS-CoV-2 in the context of groundwater contamination.

## 4. Impact of Environmental Factors on COVID-19 Transmission

Several recent studies have proposed that high temperature and humidity can play an important role in the reduction of COVID-19 infections. The low number of COVID-19 infections in Asian countries, particularly in tropical areas, is somehow documented with the explanations of high temperature and high relative humidity having a negative effect on the spread of SARS-CoV-2 [52]. It was further reported that a dried virus particle can retain its viability for over 5 days at temperatures of 22–25 °C and relative humidity of 40–50%. However, the higher relative humidity of >95% and a temperature of 38 °C significantly reduce the viability of the virus [53]. A very recent study has reported that, through high temperature and high relative humidity, the reproductive number (*R0*) of SARS-CoV-2 is significantly reduced, which in turn reduces the transmission of the disease. In another study, the reproductive number, *R0*, was calculated for each of the 100 Chinese cities with more than 40 cases of COVD-19. It has been observed that a one-degree Celsius rise in temperature and one percent increase in relative humidity lower the *R0* value by 0.0225 and 0.0158, respectively [54]. Similarly, another study has concluded that COVID-19 also decreases due to the increase in temperature [55]. These findings are consistent with the fact that other viruses like the influenza virus and SARS-CoV transmission were also significantly reduced as a result of higher temperatures and higher relative humidity [27,56,57,58].

However, it is not clear if higher temperature and humidity have a negative effect on the spread of COVID-19 because scientists around the globe have drawn contradictory conclusions. Some researchers support the claim that higher temperature and humidity can reduce the spread of COVID-19, while others oppose it [59]. After analyzing the meteorological data of 30 Chinese cities, it was concluded that low temperature, low humidity, and mild diurnal temperatures possibly help the spread of COVID-19 [45]. Another study supported this conclusion that, due to an increase in temperature, the infections of COVID-19 decrease [31]. On the other hand, another analysis of similar meteorological data of 122 Chinese cities showed a positive relationship between temperature and SARS-CoV-2 infections: a 1 °C rise in mean temperature was linked with a 4.9% increase in the daily COVID-19 cases [60]. However, further studies are required to systematically investigate the effect of environmental factors on the viability of SARS-CoV-2.

## 5. Environmental Changes Linked to COVID-19

To satisfy the needs of the growing population, urbanization and building of new industries have been very necessary, which caused detrimental effects on the environment. Consequently, the lockdown period of COVID-19 exhibited an improvement in the global environment. Some of the positive and negative aspects of COVID-19 on the environment are discussed below.

### 5.1. Decreased Air Pollution

Air pollution, being one of the most important contributors in environmental health risk, causes approximately 4.2 and 3.8 million premature deaths a year due to outdoor and indoor pollution, respectively [61]. As to control the SARS-CoV-2 transmission, government authorities across the globe have implemented a partial or complete lockdown ranging from few weeks to months. People throughout the world have been banned to move freely outside their homes to prevent the community transmission of COVID-19. Gatherings have been cancelled, various industries were banned to operate, and transport services have also been shut down. The use of conventional energy sources or fossil fuels has been considerably lowered due to the lesser demand of power in industries. Noticeably, all these measures taken to control the transmission of SARS-CoV-2 had an outstanding impact on the environment via reducing the emission of toxic greenhouse (and other) gas emissions [62]. The level of air pollution in New York almost dropped by 50% compared to the normal routine before lockdown [63]. Similarly, the data in China showed a 25% decrease in emissions during the coronavirus lockdown [64]. According to the reports of the Ministry of Ecology and Environment, the air quality was 11.4% healthier in more than 330 cities of China compared to the air quality in the last year. It has also been shown via satellite images that nitrogen dioxide (NO_2_) emission was reduced in European countries such as northern Italy, Spain, and the UK [65]. All these studies suggest that air quality can be improved by following certain control measures.

### 5.2. Alleviated Water Pollution

An improvement in water quality has also been reported during COVID-19 lockdown. In India, the effects of COVID-19 spread in the hydrosphere were evaluated by the use of remote sensing [66]. The authors selected a freshwater (Vembanad) lake as one of the most polluted lakes and found that the pollutants’ concentrations were lowered by an average of 15.9% during the lockdown compared to the pre-lockdown period. They further demonstrated that the concentrations were lower in April 2020 [66]. Due to varied anthropogenic activities, the deterioration of water quality was reported for two Indian rivers (Ganga and Yamuna) [67]. However, the COVID-19 lockdown drastically improved the surface water quality of these rivers. As per reports, the dissolved oxygen (DO) levels of the river Ganga has gone above 8 ppm and biological oxygen demand (BOD) levels down below 3 ppm, which had been around 6.5 and 4 ppm in 2019, respectively [68]. Similarly, the quality parameters of major water reservoirs (across the globe) have been reported to be improved by the COVID-19 lockdown. These results suggested that industrial pollution and tourism (during non-COVID situation) had a significant impact on water quality.

### 5.3. Elevated Mask/Gloves Pollution

One of the biggest concerns for the environment, during the COVID-19 pandemic, is the generation of medical waste, which includes masks, gloves, and other personal protective equipment(s) used by healthcare workers while dealing with COVID-19 patients. In addition, the public has been advised to wear masks and sanitize their hands as a precautionary measure and when billions of people are wearing one or two masks and/or gloves per day and then discarding, the amount of trash generated as a result would obviously be substantial. Efforts have been made to manage this huge waste, but probably are not enough to protect the environment. A survey performed by an environmental non-government organization (NGO), Ocean Asia in Soko islands, demonstrated that a huge amount of discarded single use masks washed up to a 100-m stretch of the beach in Hong Kong [64]. Because medical masks (partially made up of plastic material) and empty sanitizer bottles are persistent in the environment even after being discarded, people need to take the proactive responsibility and discard their masks and gloves at government designated waste bins [69].

### 5.4. Reduction in Noise Pollution

Noise—an undesirable sound, disturbing normal communication [70]—is considered as one of the major pollutants. Further, exposure to high level of noise for a longer period of time can lead to stress, mental disorders, irritation, sleeplessness, annoyance, loss of concentration, and hypertension [71]. During the COVID-19 pandemic and consequent lockdown situations, road traffic was very rare and the majority of industrial operations also halted. Therefore, the noise level was significantly reduced. It was further estimated in a study that the noise level in India was reduced up to 35% to 68% from 8:00 a.m. to 4.00 p.m. At Govindpuri metro station, the noise level before lockdown was 100 db and after the lockdown, it was reduced up to 50% and recorded to be 50 db only [72]. Moreover, the noise was reduced from 55 to 30–35 db in residential areas of New Delhi.

## 6. Socio-Economic Impacts of COVID-19

COVID-19 is not only causing damage to human society via killing millions of people, but also has disbanded the global economy. During the (complete/partial) lockdown, national and international flights, business activities, and transport have largely been suspended except those related to essential goods. In many countries, educational, commercial, sports, and other institutes were closed; industries were shut down for a long period of time. The tourism industry has also suffered a lot due to the current pandemic. Oil prices have also dropped significantly because major industries throughout the world were closed, which in return caused oil demand to be less. In brief, almost every sector of the global economy has severely been affected by COVID-19. Additionally, the economy of even powerful countries of the world is badly suffering [73].

COVID-19 affected different socio-economic groups in different ways. During the COVID-19 associated lockdown, demand for agricultural products from hotels and restaurants dropped, causing a 20% drop in prices of agricultural products [74]. British Plastics Federation (BPF) performed a survey to check the impact of COVID-19 on the manufacturing businesses and the results demonstrated that almost 98% of respondents admitted there was a negative impact of COVID-19 on business operations [75]. Transportation issues and staff deficiencies are key reasons for negatively inclined businesses. Moreover, COVID-19 has affected the educational system at all levels. Many countries have introduced protective measures, ranging from partial to complete closure of educational institutes. In addition, over 100 countries have implemented such closures nationwide which has affected about 900 million students [76].

COVID-19 has pushed 40–60 million people into extreme poverty (United Nations 2020). Further, people lacking the adequate social protection, internationally displaced people (IDPS), women, and slum dwellers are the most vulnerable to the COVID-19 socio-economic impacts. During lockdown, women were subjected to domestic violence which includes, physical, emotional, and sexual abuse. However, the socio-economic impact of COVID-19 is largely unknown, and assessments are being done around the world as the COVID-19 situation is unfolding. Along with the health and global humanitarian response plan, the socio-economic aspect will also be the critical component of UN’s COVID-19 response [77].

## 7. Sustainable Strategies to Reduce COVID-19 and to Prevent Future Outbreaks

**Figure 2:**
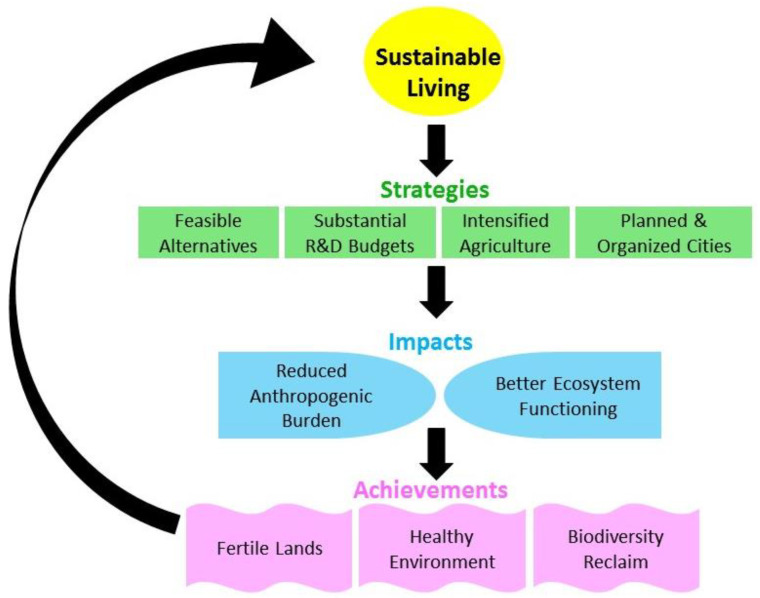
Presentation of the proposed sustainable human lifestyle to improve the environment, especially in the context of disease outbreaks such as the COVID-19 pandemic.

The COVID-19 pandemic has demonstrated that we need to strengthen our resilience to such outbreaks and other emergencies. Therefore, sustainable strategies should be followed to avoid COVID-19 like disease outbreaks causing future threats to human life and the economy. In the current scenario, authorities throughout the world are concentrating on the health system and working on the assessment of the economic losses due to the pandemic. However, in the long term, governments need to focus on the environmental aspects as well, which are very important in the potential spread of COVID-19 like diseases. In the modern world, rapid industrialization is taking place, which is very harmful for the natural biodiversity. These environmental modifications include landscape change, agriculture expansion, deforestation, urban constructions, and mining—all causing the loss of natural habitats to non-human life. Consequently, humans are occupying more and more area, in return depriving the livable area for wild animals and other life. Furthermore, such anthropogenic activities and industrialization have increased noise and water pollution [78]. Due to such changes and varying lifestyle, humans encounter wild animals and subsequently increase the risks of various zoonotic infections via their encounter with different disease-causing agents (e.g., viruses) present in the wild animal population. In this regard, long-lasting sustainable policies should be devised to control industrialization and urbanization via planned and organized cities (on grey but not on green land), adopting the green alternatives for reduced anthropogenic burden, which ultimately should achieve better ecosystem functioning and reduce noise and water pollution (Figure 2). Such strategies will serve to achieve the biodiversity reclaim and healthy environment that would ultimately be a feasible scenario for human as well as for non-humans living on this planet. Moreover, air pollution is a very important contributor in environmental health risk and so air quality improvement may benefit the resilience of people against respiratory diseases caused by viruses like SARS-CoV-2. Therefore, deforestation should be strictly banned and projects for growing new trees should be implemented by the government authorities, across the globe. Furthermore, the air polluting coal power plants should gradually be substituted with sustainably efficient green energy alternatives.

The transmission of virus can be reduced significantly if people regularly wash their hands [79], and governments were also calling for regular wash hands prevention for SARS-CoV-2 [80]. Therefore, improved access to clean drinking water and feasible sanitation facilities would avoid future pandemics and limit the spread of COVID-19. Wastewaters may have a crucial role in viral incidences within human communities. It has been recently reported that screening of SARS-CoV-2 at municipal wastewater yielded important clues about the evolution of the pandemic and even the detection of the virus outbreak in advance [81]. Very importantly, water supply strategies should ensure that wastewater is not getting mixed into the drinking water, especially in poor countries where such problems are very common.

The generation of large amounts of medical/clinical waste can harm the safe environment [82]. Such medical waste normally increases during an epidemic period and if improperly collected or treated, it can further cause the epidemic to accelerate and pose significant threats to healthcare staff, patients, and waste collection personnel. Healthcare workers are the frontline warriors against the COVID-19 pandemic, so authorities need to facilitate them in order to control future outbreaks [83]. Very importantly, medical waste contains many hazardous substances, infected masks, gloves, personal protection equipment (PPEs), and infected needles. Therefore, the safe disposal and treatment of medical waste is very important to prevent its negative impacts on human health and the environment. Strict regulations, in all countries of the world, should be implemented to ensure the proper disposal of medical and other types of waste. In addition, feasibility of degradable masks and gloves that meet the health safety standard should be evaluated and executed to overcome the increasing mask/gloves pollution.

The healthy human lifestyle should be promoted across the globe to combat future outbreaks. For instance, people should be encouraged not to use wildlife as a frequent source of food which is common in China and some other countries [84]. Instead, farm animals should appropriately be screened (before the use) for meat purposes. Public awareness, in this regard, is also very important: one of the challenges we are still facing for COVID-19 is that people are careless and so unintentionally adding fuel to the fire [84]. Therefore, the public preparedness on ‘*how to deal a challenging disease outbreak*’ is of the prime importance. Further, regular and adequate public communication of measures taken by the government should be announced to strengthen the trust of people in the government. This will also help to fight against misinformation, which is still one the biggest challenges of COVID-19. Some countries are innovating different ways of communication with public and governance through digitalization which would help to combat COVID-19. In this regard, governments should partner with the private sector to evaluate and develop sustainable, secure, and efficient ways of communication and governance in case of any future outbreaks.

The readiness of the medical system throughout the world to tackle any unexpected disease scenario is also very crucial. Unfortunately, during the COVID-19 pandemic, people across the globe have almost been fighting for masks and hand sanitizers. Instead, the world could better deal with the situation: rich countries could facilitate the provisions of the medical necessities and there should even be a mechanism where developing and/or underdeveloped (poor) countries could have access to the necessary equipment based on their infection rate to save human lives. As per scientific reports, 90% antibodies (produced in recovering COVID-19 individual) are lost in just 2–3 months’ time [85]—meaning that unless we get sustainable solutions, COVID-19 is pain for everyone. In continuity—for COVID-19 vaccines—US, Canada, and some European countries have largely been struggling to have likely solutions. However, COVID-19 disease will stay in the world unless developed vaccines are equally available to the whole world, particularly in poor countries, so the *win-win situation* would be to help everyone and to remove COVID-19 from everywhere.

## 8. Concluding Remarks/Future Perspective

During this modern era of science and technology, as a causative agent of flu and cough, the SARS-CoV-2 virus has devastated human life, not only through medical losses but also through trillions of dollars economic crunch. The disease has steadily spread across the whole globe in a matter of just 2–4 months. Various indirect dissemination factors and routes have been discussed in this review but current investigations on the environmental spread of this disease have largely been overlooked. As by the scientific definition, viruses behave like particles in the environment—outside the living organisms—and therefore the particle nature of COVID-19 should thoroughly be studied in various environmental settings. During this COVID-19 pandemic, while efforts are largely put forth for its cure and vaccines, a similar level of effort is also required for its prevalence and transmission through various environmental settings. The scientific community and policy makers should take this matter as seriously as the disease itself. The environmental media i.e., soil, air, and water are the actual beholding for the COVID-19 and all its associated challenges.

To improve the overall ecosystem functioning for humans, cutting down on unnecessary luxuries and the exploration of more viable alternative ways for an eco-friendly and sustainable lifestyle would better help safeguard the available environment. One such option is intensive agriculture, already adopted in the Netherlands, which may supply multifold food than today from even less agricultural land of the current use [86]. Intensive agriculture would allow us to dedicate the spared land for recreation and conservation purposes that may ultimately support to reclaim the biodiversity in natural environments. Briefly, substantial scientific investments and multidimensional cooperation in research, the improved worldwide health care systems, environment-friendly global policies, and regional strategies, altogether, would orchestrate better future outcomes for any human problem like the current COVID-19 pandemic.

## Figures and Tables

**Table 1:** Comparative features of the Severe Acute Respiratory Syndrome Coronavirus 2 (SARS-CoV-2) and related human coronaviruses.

	SARS-CoV	MERS-CoV	SARS-CoV-2	References
Outbreak	2002–2003	2012	2019–2021	World Health Organization
Total cases	8098	2506	124,535,520 *	
Deaths	774	862	2,738,876 *	
Fatality rate	≈10%	≈35%	2.2% *	
Basic reproduction number, R0	1.7–1.9	0.7	2–2.5	Riou and Althaus, 2020; Ashour et al., 2020
Receptors	ACE2	DPP4 (CD26)	ACE2	Li et al., 2020
Mode of action	Attachment to ACE2 receptor expressed on the epithelial cells of lungs, kidney, tongue, heart, and liver.	Binds to the pneumocystis and epithelial cells of the respiratory tract.	Binds to ACE2 receptor present on the lungs, kidney, heart, lower respiratory tract, and gastrointestinal tract.	Fani et al., 2020
Mode of spread	Originated from animals and then spread from human-to-human (Cough, sneeze, surface contact, and aerosols)	Zoonotic transmission with very little human-to-human transmission.	Zoonotic origin and then human–human spread (Cough, sneeze, surface contact, and aerosols and many others).	Azhar et al., 2014; Phan et al., 2020
Environmental implications	Not reported	Environmental contamination of hospital surfaces	Increased (medical) waste, soil and water contaminations, reduced waste recycling, huge socio-economic losses	Kim et al., 2016; Zambrano-Monserrate et al., 2020

* Data accessed on 26 March 2021. MERS-CoV, Middle East Respiratory Syndrome Coronavirus; ACE, angiotensin-converting enzyme; DPP, dipeptidyl peptidase.

## Data Availability

This manuscript has no such data to announce at public repositories.
